# Lamin A/C: Function in Normal and Tumor Cells

**DOI:** 10.3390/cancers12123688

**Published:** 2020-12-09

**Authors:** Niina Dubik, Sabine Mai

**Affiliations:** Department of Physiology and Pathophysiology, University of Manitoba, Winnipeg, MB R3E0V9, Canada; dubikn3@myumanitoba.ca

**Keywords:** lamin A/C, lamin proteins, nuclear lamina, genomic instability, tumor cells, cancer, prostate cancer

## Abstract

**Simple Summary:**

The aim of this review is to summarize lamin A/C’s currently known functions in both normal and diseased cells. Lamin A/C is a nuclear protein with many functions in cells, such as maintaining a cell’s structural stability, cell motility, mechanosensing, chromosome organization, gene regulation, cell differentiation, DNA damage repair, and telomere protection. Mutations of the lamin A/C gene, incorrect processing of the protein, and lamin A/C deregulation can lead to various diseases and cancer. This review touches on diseases caused by mutation and incorrect processing of lamin A/C, called laminopathies. The effect of lamin A/C deregulation in cancer is also reviewed, and lamin A/C’s potential in helping to diagnose prostate cancers more accurately is discussed.

**Abstract:**

This review is focused on lamin A/C, a nuclear protein with multiple functions in normal and diseased cells. Its functions, as known to date, are summarized. This summary includes its role in maintaining a cell’s structural stability, cell motility, mechanosensing, chromosome organization, gene regulation, cell differentiation, DNA damage repair, and telomere protection. As lamin A/C has a variety of critical roles within the cell, mutations of the lamin A/C gene and incorrect processing of the protein results in a wide variety of diseases, ranging from striated muscle disorders to accelerated aging diseases. These diseases, collectively termed laminopathies, are also touched upon. Finally, we review the existing evidence of lamin A/C’s deregulation in cancer. Lamin A/C deregulation leads to various traits, including genomic instability and increased tolerance to mechanical insult, which can lead to more aggressive cancer and poorer prognosis. As lamin A/C’s expression in specific cancers varies widely, currently known lamin A/C expression in various cancers is reviewed. Additionally, Lamin A/C’s potential as a biomarker in various cancers and as an aid in more accurately diagnosing intermediate Gleason score prostate cancers is also discussed.

## 1. Introduction

Lamin A/C is a protein found in the nuclear lamina of cells [1,2]. It has many roles in cells, from maintaining nuclear shape to regulating gene expression [3,4,5]. As it plays many roles in the cell, it is also involved in various diseases and cancers [6]. One cancer it has been shown to affect is prostate cancer [7,8]. Better understanding of lamin A/C’s role in prostate cancer could help with a more accurate diagnosis of prostate cancer and ensuring patients receive appropriate treatment [7,8]. 

## 2. Lamin Proteins

Lamin A/C belongs to the lamin family of proteins [1]. Lamins are 60–80 kDa proteins, which were previously thought to only be found in metazoans, however, lamin-like proteins in non-metazoans, such as NE81 found in *Dictyostelium discoideum,* have since been discovered [9,10]. Lamins come in two varieties, A-type lamins and B-type lamins [1,11]. Lamin A and lamin C are A-type lamins, commonly referred to together as lamin A/C [1,11]. Lamin A and Lamin C mRNA are produced by the same gene via alternative splicing, and, as they are quite similar, they are frequently studied together and referred to as lamin A/C [12]. While fibroblasts containing only lamin A or lamin C have a slightly abnormal nuclear shape, it appears only either lamin A or lamin C is sufficient for survival as mice made to express only lamin A or lamin C appear normal and healthy [13,14]. At least one A-type lamin is necessary for survival [13,15]. Several mouse models have been made to demonstrate this observation. The mouse model frequently used to show lamin A/C knockdown has exons 8–11 deleted, and these mice typically die 4–8 weeks after birth [15]; however, this model still expresses a truncated form of lamin A [15]. Kim et al. found that *Lmna*^Δ/Δ^ mice, which have a 357 base pair fragment of the *LMNA* gene deleted, do not express truncated lamin A/C, and these mice die even sooner, at 16–18 days after birth [15].

Lamin proteins are intermediate filaments [16,17]. Intermediate filaments are divided into six subtypes, of which lamin proteins are type V [1]. Intermediate filaments, which are part of the cellular cytoskeleton, have an average diameter of 10–12 nm, which is in between the diameters of actin and microtubules, hence the name “intermediate” [1,18]. However, at an average diameter of 3.5 nm, a lamin A/C filament is thinner than the average intermediate filament [19]. Intermediate filaments are quite sturdy and can withstand stretching and bending without being damaged; therefore, their role in cells usually has to do with cellular morphology and mechanics [18]. 

While some lamins are nucleoplasmic, most lamins in the cell are found in the nuclear lamina [12,16,17,20]. The nuclear lamina is a meshwork of A- and B-type lamins found under the inner nuclear membrane [16]. Both A- and B-type nuclear lamin proteins have many different roles in the cell. The nuclear lamina provides structural support to the nucleus, thereby maintaining proper nuclear morphology [11]. The nuclear lamina also links the cytoskeleton to the nucleoskeleton via Linker of Nucleoskeleton and Cytoskeleton (LINC) complexes, which are composed of Sad1/UNC-84 (SUN) domain proteins and Klarischt/ANC-1/Syne homology (KASH) domain proteins [11,21]. The LINC complex SUN1 and SUN 2 proteins can interact with nesprin family proteins found on the outer nuclear membrane, which interacts with the cytoplasm [1]. A- and B-type lamin proteins have many binding partners and form many interactions with various inner nuclear membrane proteins and heterochromatin and accordingly play many different roles in the cell, from chromatin organization to DNA repair [1,16,17,20]. Additionally, it has been found that depending on the tissue type, lamins have different binding partners, which could cause it to play different roles depending on the tissue type [1].

## 3. Lamin A/C: Gene, Protein, and Nuclear Lamina Structure

The human genome has three genes, which code for lamin proteins; *LMNA*, *LMNB1*, and *LMNB2* [1,17,20,22]. B-type lamins are encoded by the *LMNB1* and *LMNB2* genes. A-type lamins are encoded by the gene *LMNA,* which, along with the major isoforms A and C, also encodes minor isoforms C2 and AΔ10 [1]. Lin and Worman used sequencing and restriction mapping to determine structural organization of the human *LMNA* gene and found the gene has 12 exons, and the coding region is around 24 kb [12]. Additionally, it was found that lamin A and lamin C are identical up to the 566th amino acid [12]. Alternative splicing occurs at exon 10 to result in mRNA for prelamin A and lamin C [12]. B-type lamins have three subtypes; B1, encoded by *LMNB1,* and B2 and B3, which are encoded by *LMNB2*, with B3 being only found in amphibians and fish [1,22].

Lamins have the typical tripartite structure found in other intermediate filaments, made up of a central α helical domain with a N-terminal head and C-terminal tail on either side [1,16,17,20]. The central α helical rod domain is made up of four segments 1A, 1B, 2A, and 2B, connected by subdomains L1, L12, and L2 [1,22]. Unlike other cytoplasmic intermediate filaments, lamins also have a nuclear localization signal, and Ig fold in their tail domain [1,17,20]. The Ig domain is made up of two β sheets and plays a role in protein-protein and protein-ligand interaction [1]. Additionally, lamin A has two extra exons that are spliced out in lamin C, which encode a CAAX box (C = cysteine, A = aliphatic residue, X = any other residue) [1,12,23]. The CAAX box can be modified via farnesylation and allows lamin A’s immature form, prelamin A, to undergo post-translational modifications [1,12,16,23]. B-type lamins also contain a CAAX box, and unlike lamin A, remain permanently farnesylated [22]. As lamin C lacks a CAAX box, it is translated as its mature form and requires no further processing [23].

As *LMNA* translation and splicing produces prelamin A, which cannot incorporate into the nuclear lamina, it must be post-translationally modified to make mature lamin A [12]. To form lamin A, the cysteine of the CAAX box is first farnesylated by farnesyltransferase, then the last three amino acids of the protein are cleaved by RAS-converting enzyme 1 (RCE1) or Zmpste24, and the farnesylated cysteine is methylated by isoprenylcysteine carboxyl methyltransferase (ICMT) [16,17,23]. It is thought that farnesylation helps B-type lamins and lamin A localize to the nuclear envelope [1]. This is further shown by the fact that during mitosis, B-type lamins, which are permanently farnesylated, remain at the nuclear periphery, while lamin A, which has its farnesyl moiety cleaved, is far more soluble [22]. As lamin C has no CAAX box and, therefore, cannot be farnesylated, lamin C is able to rely on other methods localize to the nuclear envelope [1]. Besides farnesylation, lamin proteins can be targeted to the nuclear envelope via specific membrane receptors, which bind lamins [24]. Additionally, phosphorylation plays a role in lamin A/C incorporation into the nuclear lamina, as well as disassembly during mitosis [25]. It is also thought that lamin A can aid lamin C in nuclear envelope localization [24]. Once the lamin A is localized to the nuclear envelope, Zmpste24 cleaves the last 15 amino acids of the protein, including the farnesylated cysteine, to form mature lamin A [16,17,22,23]. Proper prelamin A processing is important as incorrect processing can result in various diseases [14]. For example, if the final 15 amino acids fail to be cleaved, it can lead to progerin, the permanently farnesylated mutated lamin A found in progeria, an aging disease [17]. Additionally, mutations resulting in a lack of Zmpste24 can also cause restrictive dermopathy, which results in bone and skin defects, among other complications, leading to early death in humans [13]. While *Zmpste24*^−/−^ mice do not die soon after birth like humans, they do develop muscle weakness, reduced bone density, and slowed growth [13]. 

While generally lamin A and lamin C levels are equal in cells, differences have been found in some tissues, with neuronal cells appearing to express higher lamin C levels and only lamin C being found in the human skin’s basal layer [5,23]. In mice, neuron and glia cells show a lack of lamin A, and it has been found that the brain-specific microRNA miR-9 only downregulates expression of lamin A but not lamin C in these cell types [14]. Additionally, the lamin A/lamin C ratio has also been shown to be altered in some diseases such as HIV, type 2 diabetes, progeria, and dilated cardiomyopathy [23]. Lamin C is also only found in mammals, while lamin A is found in all vertebrates [23]. Studies have also found that lamin A and lamin C have different binding affinities and partners, one example being SUN1, a nuclear envelope protein, which only binds lamin A [23]. 

**Figure 1:**
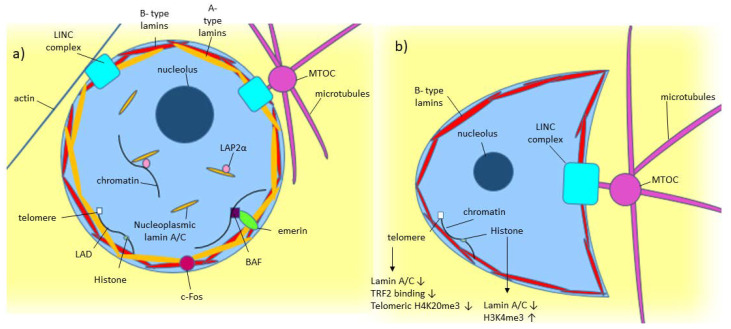
Model of known Lamin A/C functions in normal and diseased cells. (**a**) A-type lamin’s location and binding partners within the nucleus in normal cells. (**b**) Effect of low/absent lamin A/C on a cell, including crescent-shaped nuclear morphology caused by the microtubule organizing center (MTOC) pushing into the nucleus [30].

As shown in Figure 1a, lamin A/C is commonly found around the nucleus as part of the nuclear lamina, where it polymerizes to form filaments around the nucleus [1,2]. Some lamin A/C is also found in the nucleoplasm [1]. Nucleoplasmic lamin A/C is not polymerized and is more mobile [1,2,26]. While some are probably bound to structures within the nucleus, the mobile portion of nucleoplasmic lamin A is around 60% [2]. Lamin-associated polypeptide 2α (LAP2α) is a lamin binding partner, which has been found to help in localizing lamin A to the nuclear interior rather than the lamina [2].

Cryo-electron tomography (Cryo-ET) has shown that the nuclear lamina is around 14 nm thick, and the A- and B- type lamins are found in lamin filaments with alternating tetrameric and hexameric regions [19,27]. To form these tetrameric filaments, lamin proteins first form dimers via coiled-coil interactions of their rod domains [11,22]. In vitro, it has been found that different types of lamins can form heterotypic dimers, but in vivo, it has been found that lamins likely only form homotypic dimers [1,22]. These dimers then assemble end to end, head to tail, to form protofilaments in which the Ig-domains are spaced about 40 nm apart [11,19]. Finally, these protofilaments join laterally to form the final tetrameric filaments found in the nuclear lamina [11]. 

While in vitro, lamin filaments form paracrystalline arrays, in vivo, it has been shown with confocal microscopy of HeLa cells and 3D-SIM imagining of mouse embryonic fibroblasts that A- and B-type lamins instead form a meshwork [11,19,26]. In mammalian somatic cells, A- and B-type isoforms form separate meshworks in the nuclear lamina with the A-type lamin meshwork facing the nucleoplasm and the B-type lamin meshwork outside of the A-type mesh [5,16,19,26,27]. The meshworks are thought to be separate, but the silencing of one isoform has been shown to affect the meshworks of the remaining isoforms. For example, silencing of lamin B1 led to the enlarging of the lamin A/C mesh [11,26]. Lamin B1 silencing also caused nuclear blebs, found to contain only lamin A/C and no B-type lamins [11,22,26]. It was suggested that this phenomenon occurred because lamin B1 is found outside the lamin A/C meshwork, holding it in, and, therefore, its loss causes the lamin A/C meshwork to expand, causing blebs [5]. As silencing one type of lamin was shown to affect the remaining lamins in the nuclear lamina, it is likely that the different lamin isoforms still interact with each other despite forming separate meshworks [19,26,27]. When it comes to how lamin A is incorporated into the nuclear lamina, one study found that Sorting Nexin 6, which regulates the trafficking of various proteins, plays a role in lamin A import to the nucleus using a RAN-GTP (Ras-related nuclear protein) gradient [28].

During mitosis, the nuclear lamina must disassemble. This is achieved through phosphorylation of the lamina [26]. CDK 1 phosphorylates the lamins during mitosis, and it has been found that A-type lamins leave the nuclear lamina first and are freely diffusible in the nuclear interior, unlike B-type lamins, which remain near the nuclear periphery even after phosphorylation [26]. Protein phosphatase 1 dephosphorylates the lamins when reassembly of the lamina is needed [26]. In addition to phosphorylation, lamins can also undergo various other modifications such as sumoylation, acetylation, and ubiquitylation [1,2]. Lamin A/C acetylation has been found to be important for lamin A/C to function correctly. MOF, a lysine acetyltransferase, which is part of the non-specific lethal (NSL) protein complex acetylates lamin A/C [29]. In MOF knockout mouse MEFs, loss of lamin A/C acetylation results in lamin A/C having increased solubility, which greatly affects its functioning in mechanotransduction and resulted in nuclear blebbing, nuclear envelope rupture, transcriptional defects, and general genomic instability [29].

## 4. Lamin A/C: Role in Structural Stability

Lamin A/C plays many important roles in the cell. One of these roles is in maintaining structural stability of the cell. Particularly lamin A/C in the nuclear lamina increases nuclear stiffness while still allowing elasticity, which helps maintain proper nuclear morphology even under mechanical stress [4,5]. The linker regions of the rod domain of lamin protein are flexible and have been shown to allow the rod domain of the protein to contract [31]. Additionally, the head and tail domain of lamin proteins form electrostatic interaction with each other in lamin tetramers, which can be broken by pulling forces, allowing the lamin filament to stretch, lending further elasticity to the lamin filament [31]. This flexibility allows lamin filaments to undergo mechanical forces without negative effect, and mutations in lamin’s rod domain have been shown to lead to various pathologies [32,33]. This has been further shown in the nuclei of *Lmna*^−/−^ mice fibroblasts, which tend to be more fragile and deform easily under mechanical stress [1,34]. Additionally, lamin A/C knockdown has been shown to result in crescent-shaped nuclei, as seen in Figure 1b [30]. This is because radiating microtubules push the microtubule organizing center (MTOC) into the nucleus in normal cells, but lamin A/C’s structural support prevents nuclear deformation [30]. In lamin A/C knockdown cells, the nucleus is not stiff enough to resist the MTOC’s force, hence leading to crescent-shaped nuclei [30]. The molecule remodlin restores nuclear shape in Hutchinson–Gilford progeria syndrome (HGPS) cells, which contain a mutated form of lamin A/C called progerin, as remodlin inhibits N-acetyltransferase 10 (NAT10), which is linked with SUN1 and plays a role in anchoring microtubules [35]. Therefore, inhibiting NAT10 stops MTOC forces on the nucleus [35,36].

As well as microtubules, lamin A/C interacts with actin via the LINC complex [37]. The LINC complex connects the nucleus to the actin cap, which is found on top of the nucleus during interphase in many adherent somatic cells and protects these cell’s nuclei from mechanical deformation [30,37]. This is especially important for cells in tissues that are frequently under mechanical stress, such as skeletal and cardiac muscle tissue, thus that they can maintain their shape and integrity [38]. 

## 5. Lamin A/C: Role in Mechanosensing, Mechanosignalling, and Cell Movement

As well as contributing to structural stability, lamin A/C is also involved in mechanosensing and mechanosignalling [5]. As it links the nuclear lamina with the cytoskeleton, the LINC complexes play roles in mechanosensing as well as nuclear migration and nuclear position [1]. This allows the nucleus to sense the surrounding environment and change the proportion of lamin A/C in the periphery and nucleoplasm as well as the extracellular matrix composition to stiffen or soften the nucleus depending on the cell’s external environment [2,4,22]. This ability to sense the cell’s environment plays an important role in cell differentiation [21,39].

The LINC complex plays an important role in cell polarization and motility. This can be shown in wound healing. In wound healing, the MTOC repositions to face the side of the cell nearest to the wound [40]. This can only be done if the MTOC is properly connected to the nucleus, which is a function of lamin A/C via the LINC complex [40]. Therefore, *Lmna*^−/−^ fibroblasts show slowed wound healing as MTOC does not position correctly [40,41]. Additionally, in wound healing, the cytoplasm of fibroblasts must stiffen thus that they can move to the site of the wound [40]. As lamin A/C is responsible for cell stiffness, cells lacking lamin A/C have reduced mobility [40,41]. The LINC complex’s interaction with microtubules has further effects on T cell stimulation [41]. Upon antigen recognition, lamin A/C is upregulated in T cells [41]. This is thought to be because, in T cell stimulation, the MTOC must reposition at the T cell/antigen-presenting cell site of contact for proper T cell stimulation and, like in wound healing, the LINC complex is needed for proper MTOC orientation [40,41]. 

## 6. Lamin A/C: Role in Genomic Organization and Stability 

As well as lamin A/C’s structural roles in maintaining nuclear morphology, lamin A/C also plays an important role in genome dynamics, expression, regulation, and genome stability. A major role of lamins is in binding and organizing chromatin [42]. Typically, heterochromatin is found near the nuclear periphery, while more transcriptionally active areas of the genome are closer to the center of the nucleus [3,22,43]. Lamin binding receptor B (LBR) and lamin A/C are both necessary to maintain peripheral heterochromatin, with LBR typically being expressed early on in cell differentiation, then later being replaced by lamin A/C in differentiated cells [42]. Cells lacking both LBR and lamin A/C have heterochromatin in the nuclear interior instead [42]. Emerin, a LEM (LAP2, emerin, MAN1 domain) protein, which interacts with lamin A/C and requires lamin A/C for correct localization, anchors heterochromatin to the nuclear periphery via barrier to autointegration factor (BAF) [2,17,22,32,43,44,45]. Lamin A/C and emerin also associate with nuclear myosin 1 (NM1), which plays a role in maintaining chromosome territories in their correct positions [46]. Therefore, loss of lamin A/C and emerin results in increased chromatin mobility and loss of chromatin territories [46]. Additionally, areas of chromatin that associate with lamin A/C are called Lamin A-associated binding domains (LADs), and genes found in these areas are typically transcriptionally inactive [1,2,3]. Additionally, lamin A/C can play a role in recruiting either transcriptionally permissive or repressive histone modifications. Therefore, it is thought lamins play a role in controlling gene expression [3]. While lamin A/C plays a role in heterochromatin tethering and gene repression, not all LADs are found at the nuclear periphery, and nucleoplasmic lamin A/C and LAP2α have been shown to organize chromatin within the nucleus [17,22]. Additionally, in the nuclear interior, lamin A/C binds euchromatin as well as heterochromatin [2]. Lamin A also plays a role in genome dynamics. Typically chromatin loci show slow diffusion through chromosome territories, but it has been shown in *Lmna*^−/−^ MEFs chromosome loci diffusion is faster [47].

Lamin A/C also plays a role in DNA damage, repair, and apoptosis. Progerin causes cells to be more sensitive to DNA damage, as cells with progerin show delayed localization of p53-binding protein factor to sites of DNA damage, increased double-strand break marker γ-H2AX, and defective localization of DNA damage regulators ATR and ATM and repair factors Rad 50 and Rad 51 [1,6]. Lamins are also apoptosis targets and apoptosis is delayed in cells with lamin mutants, which are missing the site cleaved in apoptosis [1,35]. Lamin A/C also plays a role in heat shock. Hsp70 protein binds and protects DNA in elevated temperatures and lamin A/C has been found to cause Hsp70 gene locus repositioning to the nuclear interior thus, it can be transcribed after heat shock [48].

Additionally, lamin A/C interacts with telomeres. Telomere loss plays a role in cellular aging, therefore, it is thought that lamin A/C’s involvement with telomeres may explain why lamin A/C mutations can cause accelerated aging diseases [49]. Lamin A/C plays many roles in maintaining telomeres and loss of lamin A/C results in altered telomere distribution, shortening of telomeres, and defects in telomere heterochromatin [50]. 

Typically in mammals, telomeres are found throughout the nucleus in G0, G1, and S phases of the cell cycle [50,51]. In *Lmna*^−/−^ MEFs, the telomere positioning shifts closer to the nuclear periphery compared to control *Lmna*^+/+^ MEFs, suggesting that lamin A/C helps keep telomeres in their correct positions in the nucleus [50]. Additionally, *Lmna*^−/−^ MEFs have shorter telomeres than controls, showing lamin A/C has a role in maintaining telomere length [50]. Telomere repeat-binding factor 2 (TRF2) is part of the telomere-capping shelterin complex, which regulates telomere length [49,50]. TRF2 binds telomeric DNA to form t-loops, which play a role in telomere protection [49]. Lamin A/C stabilizes the binding of TRF2 in the formation of interstitial t-loops and reduced lamin A/C results in reduced TRF2 binding [49]. Additionally, progerin does not interact with TRF2, and, perhaps, as a result, cells from patients with HGPS have shortened telomeres [36,49]. Telomeric heterochromatin also regulates telomere length, and it has been found that histone marks are altered in both HGPS fibroblasts and *Lmna*^−/−^ MEFs, with *Lmna*^−/−^ MEFs having reduced telomeric H4K20me3 [50]. 

As seen previously, lamin A/C plays a role in DNA damage repair. This also affects telomeres. Dysfunctional telomeres are processed via non-homologous end joining (NHEJ), and loss of lamin A/C affects this process [50]. Dysfunctional telomeres activate the DNA damage repair pathway, and knockdown of lamin A/C affects various cellular components involved with DNA damage repair, such as increased γ-H2AX and decreased 53BP1 [50]. Overall, this results in cells lacking lamin A/C being unable to properly process dysfunctional telomeres [50]. 

## 7. Lamin A/C: Role in Gene Expression and Regulation 

Lamin A/C is associated with many genes and signaling pathways. Lamin A/C has been shown to interact with many promoters, and it is thought that its positioning affects whether the gene is repressed, with positioning upstream proximal of the transcriptional start site repressing gene expression, while upstream distal causes increased expression [3]. Additionally, as well as its role in mitosis, phosphorylated lamins can also stay in the nuclear interior, where they play a role in gene regulation by binding enhancers on euchromatin [52]. Lamin A/C can also affect gene expression by affecting histone post-translational modifications, with lamin A/C knockdown resulting in a global increase of transcriptionally permissive H3K4me3 enrichment even on genes not directly associated with lamin A/C [3]. Mutations in lamin A/C have also been found to inhibit RNA polymerase II and prevent initiation factor TATA-binding protein from properly localizing, thereby preventing transcription [1,26,34,48]. Lamin A/C affects various transcription regulators such as pRb and cFos and has been found to be involved in many different signaling pathways in the cell [1,4]. For example, in AP1 (Jun/Fos) regulation, lamin A/C sequesters c-Fos at the nuclear envelope until phosphorylation causes c-Fos to be released, thus, it can play its part in AP1 immediate-early gene activation [1]. 

Lamin A/C is primarily found in differentiated cells. It was previously thought that pluripotent cells completely lacked lamin A/C, explaining their irregular shape, but mouse embryonic stem cells have been found to express low levels of lamin A/C [53]. It is possible the levels are low enough for lack of lamin A/C to still account for stem cells’ irregular nuclear shape [53]. Regardless, lamin A/C does appear to play a role in cell differentiation [21]. As mentioned earlier, cells typically express LBR first, and then, as the cell progresses through differentiation, LBR heterochromatin tethering is replaced by lamin A/C [42]. This shows that lamin A/C is linked to differentiated cell states, while LBR is linked to undifferentiated cells [42]. Disrupting either LBR or lamin A/C heterochromatin tethering has been shown to affect muscle-related gene expression in differentiating myotubes with LBR deletion resulting in increased muscle-related gene expression and lamin A/C deletion having the opposite effect [42]. Additionally, increases in lamin A have been associated with tissues differentiating into stiffer tissue types [22,38,39]. Lamin A/C’s mechanosensing function plays a role in differentiation as it can sense the cell’s external environment and can differentiate accordingly, with a stiff environment increasing osteogenesis and a soft environment increasing adipogenesis [21,39]. Lamin A/C is also required for correct subnuclear localization of polycomb group (PcG) proteins [54]. PcG proteins are epigenetic repressors, which prevent premature cell differentiation, and lamin A/C downregulation results in increased muscle differentiation in myoblasts and myotubes [54].

## 8. Lamin A/C in Disease and Cancer

### 8.1. Laminopathies

As lamin A/C has so many different functions, mutations impairing these functions can lead to a wide variety of diseases. Diseases involving lamin proteins are called laminopathies and can be primary or secondary [6,13,55] Primary laminopathies are caused by mutation of the *LMNA* gene, and secondary laminopathies are caused by Zmpste24 defects, which prevents prelamin A from successfully being converted to lamin A [6,13,55]. Secondary laminopathies can also result from mutation of other non-lamin genes that interact with lamin such as lamin binding proteins like emerin [6,13,55]. Additionally, while it is lamin A/C mutations that most commonly cause laminopathies, lamin B can also be involved, with Pelger-Huet anomaly and Greenberg skeletal dysplasia being caused by a mutation to the LBR gene [22]. Laminopathies can be caused by lamin A/C mutations affecting both the cell’s structural integrity and genomic stability [56,57]. Generally primary laminopathies can be sorted into 4 types; striated muscle disorders, lipodystrophic syndromes, peripheral neuropathy, and accelerated aging diseases [6,22]. Secondary laminopathies have caused mandibuloacral disease, progeroid-like disease, and restrictive dermopathy [13].

The first disorder lamin A/C was ever found to be implicated in was Emery-Dreifuss muscular dystrophy (EDMD) [6]. EDMD primarily affects skeletal muscle, but cardiac muscle can also be affected [6]. *LMNA* mutations can also lead to dilated cardiomyopathy (DCM). DCM is the third leading cause of heart failure in the United States, and about 6% of cases are caused by *LMNA* mutations [6,56]. DCM caused by the inheritance of *LMNA* mutations is usually autosomal dominant and typically associated with a more severe phenotype and poorer prognosis than DCM caused by other causes [58]. 

Lipodystrophic syndromes cause loss of adipose tissue and insulin resistance [55,59]. As well as lipodystrophic syndromes, lamin A/C is also implicated in obesity-induced insulin resistance [60]. Lamin A/C increases pro-inflammatory NF-κβ transcription factor transcription, which mediates adipose tissue macrophage inflammation and can lead to insulin resistance and type 2 diabetes [60].

*LMNA* mutations can also cause accelerated aging disorders like Hutchinson-Gilford progeria syndrome (HGPS) and Werner syndrome [55]. 90% of all HGPS is caused by a single base-pair substitution at exon 11 of lamin A, which results in the permanently farnesylated protein progerin [13,55]. Typically the brain is not affected in HGPS patients, but it has been found that *LMNA* expression in the hippocampus increases in late-stage Alzheimer’s [61]. It is also thought lamin A/C may play a role in regular aging as many of the nuclear defects found in HGPS cells are the same as ones found in cells from older people [61,62].

Currently, laminopathies lack specific treatment. Lipodystrophic syndromes caused by *LMNA* receive the same treatment as diabetes Type II or dyslipidemia, such as diet, exercise, metformin, and insulin [59]. Additionally, recombination leptin therapy can also relieve some of the symptoms of lipodystrophy, like insulin resistance [60]. As for DCM, treatments are the same as non-*LMNA* caused heart failure and usually involve pacemakers and heart transplants once the disease becomes severe enough [6]. For HSPG, there have been some studies and trials testing farnesyl-transferase inhibitors such as lonafarnib to alleviate problems in HGPS cells, and while lonafarnib has been shown to improve nuclear shape in HGPS cells, it is too toxic for long term treatment [6,36,55]. Overall, most current treatments for laminopathies focus on treating symptoms rather than the root cause.

### 8.2. Cancer

Cancer cells have dysregulated gene expression, alterations in signaling pathways, overall genomic instability, and abnormal nuclear shape [63,64]. As lamin A/C plays many roles in the cell, including regulating gene expression, participating in signaling pathways, and maintaining proper nuclear shape, it is likely lamin A/C also plays a role in the development and/or maintenance and propagation of cancer cells. Cancer cells frequently have irregular lamin A/C expression and, as seen in Table 1, Table 2 and Table 3, lamin A/C can be over or under-expressed in many different cancer types. Additionally, lamin A/C also shows incorrect cytoplasmic localization in some cancer cells as well as abnormal internal lamin A/C structures within the nucleus [63,65,66,67,68]. However, since lamin A/C plays so many different roles in cells and its function can vary across different tissue types, its effect in cancer, as shown by Table 1, Table 2 and Table 3, is highly variable across different cancer subtypes, and there is no overall expression pattern for lamin A/C in cancer [63,67,69]. Additionally, while lamin A/C expression levels are often measured together, some studies have found lamin A and lamin C expression is not always affected equally in some cancers, further complicating lamin A/C’s effect on cancer [70,71,72].

## 9. Deregulated Lamin A/C Expression in Cancer Cells

As seen in Table 2, lamin A/C is downregulated in some cancers. Various mechanisms have been suggested as to how this occurs in different cancers. In lymphoma, leukemia, and neuroblastomas, the *LMNA* gene is silenced via hypermethylation of the CpG island promoter, and demethylating agents have been shown to increase lamin A/C levels in neuroblastoma cells [65,94,95]. In some ovarian cancer cells, caspase-6 is overexpressed, and lamin A/C is underexpressed, thus it is thought caspase-6 may play a role in degrading lamin A/C in these tissues [96]. Additionally, phosphorylation during interphase may mark lamin A/C for degradation in some cancers, particularly in cervical cancer [89,97]. It is thought that HPV infection can also result in lamin A/C degradation [89]. 

As shown in Table 1, Table 2 and Table 3, while lamin A/C expression is frequently reduced in cancer, overexpression is common in many cancers as well, and lamin A/C has been found to lend helpful characteristics to a variety of cancers. For example, as lamin A/C plays such an important role in maintaining a cell’s structure, it has been found that lamin A/C can help strengthen circulating tumor cells (CTCs) from stresses they encounter while in circulation [75]. Lamin A/C has been shown to also play a role in allowing CTCs to reattach and start new tumors in new locations [80]. Additionally, in colorectal and prostate cancer cells, lamin A/C has been found to increase cell motility, therefore, allowing cancer to invade surrounding tissue better and grow [67,81].

## 10. Nuclear Morphology and Cancer

Lamin A/C plays an important role in maintaining proper nuclear morphology, and one of the most obvious effects of depleting lamin A/C is deformed nuclear morphology [1,11,34,97]. Cancer cells also show abnormalities in nuclear morphology and, generally speaking, more nuclear deformity equals greater malignancy [93,97]. As seen in Table 2, small cell lung carcinomas (SCLC) have low lamin A/C expression, unlike non-SCLC. This reduced lamin A/C expression could explain why SCLC biopsies tend to be smaller and deformed since reduced lamin A/C may lead to more easily deformed cells [85]. Additionally, Capo-chichi et al. showed that mammary epithelial cells, which had their lamin A/C levels downregulated, showed nuclear deformities similar to breast cancer cells, and Alhudiri et al. found that breast cancer tumors with reduced lamin A/C levels resulted in poorer prognosis than tumors with high lamin A/C expression [83,93]. Therefore, in cancers where lamin A/C is downregulated (see Table 2 for examples), it is possible that abnormal nuclear shape is due to a lack of lamin A/C, and this increased nuclear deformity could result in a worse prognosis [63,83,93,97]. 

## 11. Cell Motility/Migration and Cancer

Lamin A/C can affect cell motility and plays a role in cell migration during wound healing [40,41]. Likewise, lamin A/C expression has been found to affect cell motility in some cancer types, which can cause increased metastasis. For example, colorectal cancer with increased lamin A/C expression is associated with much higher mortality rates, which is thought to be due to lamin A/C causing upregulation of actin-bundling protein, T-plastin, and downregulation of cell adhesion molecule, E-cadherin, which results in increased motility and, therefore, greater metastasis and poorer prognosis [63,65,67,76]. Another way lamin A/C expression can increase a cancer’s metastatic potential is by protecting the nucleus from mechanical stress [75,98]. When a cancer is metastatic, circulating tumor cells from the main tumor must be able to withstand mechanical stress encountered in the bloodstream in order to establish new tumors [75]. As shown in Table 1, knocking out lamin A/C in breast cancer cell lines resulted in increased apoptosis of the cells in response to fluid shear stress [75]. Therefore, as lamin A/C lends mechanical stability to the nucleus, lamin A/C upregulation in some cancers could allow circulating tumor cells from these cancers to withstand fluid shear stress in circulation better, leading to more effective metastasis [4,5,75]. However, as Table 1 shows, while lamin A/C expression causes increased motility and invasiveness in some cancer cells, such as colon adenocarcinoma cell line SW480, this does not hold true for all cancer cells. In the lung cancer cell line A549, lamin A/C upregulation decreases cell motility [67]. 

Additionally, Kaspi et al. found that, rather than upregulation, loss of lamin A was possibly related to loss of epithelial to mesenchymal transition (EMT)-involved epithelial membrane antigen (EMA)/MUC-1 in lung adenocarcinomas. EMT increases cell motility, therefore, loss of lamin A/C could be involved in EMT, and subsequently increase motility and metastatic potential in cancer cells [72]. In neuroblastoma cell lines, lamin A/C knockdown, rather than upregulation, results in increased migration of neuroblastoma cells [63]. One way downregulation of lamin A/C could increase migration is that reduced lamin A/C expression could result in a softer nucleus that could deform more easily and, therefore, migrate through tighter spaces while in circulation, than cells expressing higher lamin A/C levels [75,98]. 

Overall, either too high or too low levels of lamin A/C can negatively impact a cancer cell’s metastatic potential. Wang et al. showed this by comparing the migration rate through a 3 μm pore in the H08910 ovarian cancer cell line with overexpressed, moderately inhibited, and very inhibited lamin A [77]. As shown in Table 1, the cells migrated most when lamin A/C was 30–40% inhibited. This is because the nuclei were too stiff to pass through the pore when lamin A was overexpressed but, when underexpressed too much, the nuclei became very fragile and very easily damaged, leading the cells to have micronuclei formation and DNA damage, leading to increased genomic instability, after passing through the pore [77]. Overall, moderately inhibiting lamin A/C allowed the cells to be deformable enough to easily pass through the pore while still having enough plasticity not to be permanently damaged, showing how both over and underexpression of lamin A can lead to decreased metastatic potential in cancer cells [77]. Additionally, Zhang and Lv found that in breast tumor cell lines, lamin A/C was upregulated when the cells were suspended, but lamin A/C levels went back down once the cells reattached, showing lamin A/C may play a role in the reattachment of circulating tumor cells at new sites [80].

## 12. Cell Differentiation and Cancer

Generally speaking, the more poorly differentiated a tumor is, the poorer the prognosis [67]. Lamin A/C plays a role in cell differentiation and is found in differentiated cells, while it has only low expression in stem cells [21,22,38,39,53,64,99]. Additionally, lamin A/C expression is often lowered or absent in highly proliferating cells [79]. Jansen et al. found that in reactive lymph nodes, A-type lamins were not expressed in samples, which also expressed proliferation marker Ki67, and that lamin A/C expression in Reed–Sternberg and Hodgkin cells could indicate these cells had a more differentiated phenotype [92]. Overall, cells, which lack lamin A/C can have a more stem cell-like phenotype, which is shown in several tumor subtypes, such as squamous cell carcinoma, gastric carcinoma, and neuroblastoma, where lamin A/C downregulation results in poor differentiation and a more stem cell-like phenotype [67,79,87]. Therefore, lamin A/C could potentially be used to determine the differentiation state of tumors and, therefore, help determine the cancer’s prognosis [67]. 

Additionally, rather than downregulation, lamin A/C expression can lead to a more stem cell-like phenotype in some cancers. Lamin A/C is not expressed in colonic crypt cells except in colonic epithelial stem cells at the base of the crypts [76]. It is possible that lamin A/C expression in colorectal cancer causes these cancer cells to become more stem cell-like, therefore, leading to an increase in mortality found in lamin A/C expressing colorectal cancers [76].

## 13. Lamin A/C: Genomic Instability and Senescence in Cancer

Downregulation of lamin A/C can also cause genomic instability. Cancer cells frequently show aneuploidy [83,100]. Reduced lamin A/C can cause mitotic failure and cell cycle defects, which can lead to aneuploidy [94,100]. For example, mammary epithelial cells, which had lamin A/C downregulated became polyploid [83]. Additionally, cells with downregulated lamin A/C frequently show nuclear budding, which can lead to micronuclei formation and aneuploidy [100]. 

Lamin A/C plays a role in DNA repair [1,6,94]. Lamin A/C plays a role in both DNA double-strand break repair and DNA base excision repair, and lamin A/C knockout MEFs showed increased DNA damage [94,101]. This is relevant to cancer development as defective DNA repair can lead to mutations, which can eventually lead to genomic instability, malignant transformation, and cancer [101]. In ovarian cancer cell line H08910, when lamin A was 70–80% inhibited, the cells had downregulated BRCA1, Ku80, and Rad50, which are genes involved in DNA double-strand break response and repair [77]. Additionally, under mechanical stress, these cells expressed γ-H2AX and increased DNA damage, which lead to overall genomic instability [77]. Due to an impaired ability to repair DNA, cells with downregulated lamin A/C are also more radiosensitive, meaning it is possible that cancers with lower levels of lamin A/C might respond better to radiation therapy [94].

Lamin A/C is involved in telomere maintenance [49]. Many cancer cells produce telomerase, which allows them to avoid telomere shortening, which would eventually halt cell growth [102]. While 80–85% of tumor cells express telomerase, somatic cells express little to none, making telomerase inhibitors a good potential treatment for cancer [103,104]. Many telomerase inhibitors have been found, and they can be synthetic, or they can be isolated from a variety of bacteria, fungi, and plants such as telomestatin, which was isolated from the bacteria Streptomyces anulatus [103,104]. One example of a synthetic telomerase inhibitor is GRN163L [103,105]. GRN163L is in a clinical trial for treating various cancers, including chronic lymphocytic leukemia and non-small cell lung cancer [103,105]. Additionally, some telomerase inhibitors are synthetic derivatives of naturally occurring compounds like MST-312, a synthetic derivative of epigallocatechin gallate [103]. 

Telomerase inhibitors can cause senescence and apoptosis in cancer cells, which express telomerase [102,105]. Understandably, cells with longer telomeres are more resistant to such compounds [102]. However, lamin A/C overexpression has also been found to confer resistance to such compounds, while low lamin A/C expression causes greater sensitivity to these kinds of compounds, despite telomere length. For example, some cells like SLT-type DMS114 are sensitive to MST-312 despite having long telomeres due to their low lamin A/C expression [102]. Therefore, due to lamin A/C role in stabilizing telomeres, high lamin A/C expression many be able to help cancer cells resist telomerase inhibitors [49,102]. 

Lamin A/C can be involved in cellular senescence, which stops cell division and, therefore, prevents tumor growth [74,99]. Generally, cells that have undergone senescence have higher lamin A/C levels [99]. In several cancers, increased nestin, a type VI intermediate filament, has been correlated with poorer prognosis [73]. Nestin can interact with lamin A/C and, as shown in Table 1, nestin knockdown in lung cancer cells resulted in increased lamin A/C ubiquitination, increased cytoplasmic lamin A/C, and increased lamin A/C phosphorylation, leading to abnormal nuclear shape and senescence [73]. It is thought that nestin helps stabilize lamin A/C in tumor cells, which helps protect them from undergoing senescence, resulting in a poorer prognosis [73]. Focal adhesion kinase (FAK) was also found to be involved in senescence in tumor cells as inactivating FAK in lung cancer cells leads to downregulation of lamin A/C and senescence [106].

Lamin A/C mutations can also induce tumor cells to undergo senescence. HGPS causes accelerated aging but does not cause cancer, despite cancer risk increasing with age [74]. Moiseeva et al. looked into whether or not progerin, the mutated form of lamin A/C that frequently causes progeria, could inhibit cancer growth [74]. They found that while progerin could only induce senescence in normal cells, S22A-progerin, which had an additional mutation, which prevented phosphorylation at serine 22, did cause cellular senescence in tumor cells (Table 1*)* [74]. Since phosphorylation causes lamin A/C to disassemble from the nuclear lamina during mitosis, S22A-progerin, which could not be phosphorylated, formed a thick layer around the nucleus, which interfered with mitosis, leading to cell cycle arrest and senescence [74]. Additionally, this senescence did not involve the p53 pathway, which is frequently inactivated in tumor cells. Therefore, lamin A/C mutations could potentially play a role in cancer treatment [74]. As well as mutated lamin A/C, Matralis et al. found that inhibiting Zmpste24, which prevents prelamin A from being correctly processed, results in a similar accumulation of farnesylated prelamin A, also resulting in impaired mitosis and senescence [107].

## 14. Lamin A/C Binding Partners and Cancer

Lamin A/C interacts with many different proteins and parts of the cell, and these binding partners can also be affected in cancer cells [1,16,17,20]. It has been found that both lamin A/C and Lap2α interact with tumor suppressor pRb, and Lap2α is upregulated in some cancers [108]. Therefore, it is thought that both lamin A/C and Lap2α levels can affect pRb’s function in cancer [87,108]. Emerin also plays a role in cancer [78]. Reis-Sobreiro, et al. found that depleting diaphanous-related formin 3 (DIAPH3) or lamin A/C in DU145 and BT-549 cell lines resulted in mislocalization of emerin and a more aggressive, malignant cancer phenotype [78]. Additionally, proteins from the LINC complex have also been found to be downregulated along with emerin and lamin A/C in some cancers [84,97].

## 15. Prostate Cancer

Prostate cancer is one of the most common cancers in men, and its incidence increases with age, with 67 being the mean age at which it is diagnosed [7,109]. Prostate-specific antigen (PSA) levels can be used to detect prostate cancer, and the Gleason score is used to determine the severity [7,109]. The Gleason score is a measurement of the tumor’s differentiation, with poorly differentiated tumors having a higher score than well-differentiated ones [7,8,109]. Generally, poorly differentiated, higher Gleason score cancers have poorer outcomes [8]. The main treatments of prostate cancer are radical prostatectomy and radiotherapy [7,109]. However, these treatments may not be necessary in all cases as low Gleason score prostate cancers are frequently asymptomatic [8]. In these cases, the cancer is simply monitored to ensure it does not become more severe [7,8,109]. 

One problem that arises with the diagnosis and treatment of prostate cancer is that PSA levels and Gleason score are not perfect at determining prostate cancer’s prognosis [7,8]. Particularly for prostate cancers with an intermediate Gleason score, it can be hard to determine the cancer’s prognosis. Therefore, these cancers are prone to over or under treatment [7,8,110]. Finding biomarkers to help determine the severity of prostate cancer can help better determine appropriate treatment, and various studies have looked into lamin A/C as one such potential biomarker [7,8]. 

## 16. Deregulation of Lamin A/C and Interactive Partners in Prostate Cancer

Some studies have found that lamin A/C expression is increased in higher Gleason score prostate cancers [81,91]. Studies have found that lamin A/C expression is decreased in low and intermediate Gleason score prostate tumors but increases in higher-grade tumors [81,91]. Kong et al. also found that lamin A/C expression was heterogeneous in high-grade prostate tumors, with the center sections of the tumor having lower expression than the periphery and areas where the tumor was invading surrounding tissue [81]. This suggests that lamin A/C may play a role in prostate cancer’s ability to spread and could help differentiate high-risk tumors from low-risk tumors [81]. As seen in Table 1, this is reflected in prostate cancer cell lines LNCaP, DU145, and PC3, where upregulating lamin A/C leads to increased cell growth and motility.

P300 is a transcriptional coactivator whose expression is correlated with more aggressive prostate cancer and changes in nuclear morphology in prostate cancer cells [111]. Lamin A/C plays a large role in maintaining nuclear morphology, and when p300 was transfected into prostate cancer cell line C4-2, lamin A/C was upregulated [111]. Therefore, it is possible that lamin A/C plays a role in the increased aggressiveness of cancers expressing p300 [111].

While increased lamin A/C appears to increase prostate cancer’s aggressiveness, some studies have found the opposite: Lower lamin A/C levels are associated with increased metastasis and worse prognosis [7,78,112]. *LMNA* has been found to be downregulated in castration-resistant prostate cancer and, particularly in tumors with a Gleason score over 6, low lamin A/C has also been found to be associated with greater lymph node metastasis, increased chance of recurrence, and lower chance of survival [7,78]. One explanation for this is that lower lamin A/C may lead to softer, more easily deformable cells, which can more easily move through tight spaces to metastasize [112]. Using highly metastatic cell lines PC3 and Cl2, moderately metastatic DU145, and non-metastatic LNCaP Khan et al. showed that PC3 and CL2 were less stiff than DU145 and the control, normal human prostate epithelial cell line RWPE-1 [112]. Khan et al. found that while other factors, such as chromatin condensation state are involved, to a certain extent, lamin A/C levels are related to nuclear stiffness, with the control having the highest lamin A/C level and stiffness and PC3 and LNCaP have the least lamin A/C and least stiffness [112]. Unexpectedly LNCaP had the softest nuclei despite being non-metastatic [112]. One explanation for this was that nuclear stiffness and lamin A/C levels have to do with the location the cells metastasize to [112]. While non-metastatic in mice, LNCaP comes from lymph node metastases [112]. Cells undergo less shear stress in lymphatic capillaries than in the bloodstream, which is where the metastatic cell lines would have to travel through to arrive at their site of metastasis [112]. Therefore, as discussed previously, while lower lamin A/C aids in metastasis to an extent, too low a level result in cells too fragile to resist the shear stress they would encounter in the bloodstream [77,112].

Emerin, which interacts with lamin A/C can also affect metastatic potential and nuclear shape in prostate cancer cell lines with emerin knockdown in DU145 cells resulting in greater metastatic potential [78]. Additionally, CTCs from prostate cancer patients were shown to have reduced emerin expression [78]. Therefore, it is possible that loss of lamin A/C could potentially cause emerin mislocalization, therefore, leading to nuclear abnormalities and increased motility [78].

## 17. Lamin A/C: Role in Genomic Organization, Expression, and Instability in Prostate Cancer

Lamin A/C is involved in signaling pathways that play a role in prostate cancer. One example is the PI3k-PTEN-Akt survival pathway [81]. As seen in Table 1, in LNCaP, DU145, and PC3 cell lines, lamin A/C overexpression resulted in increased PI3k subunit p110 and p85 expression. Since overexpression of lamin A/C in these cell lines also resulted in increased cell growth and invasive properties, it is possible that lamin A/C stimulates the PI3k-PTEN-Akt pathway leading to more aggressive cancer (Table 1).

Lamin A/C also appears to be involved in epithelial to mesenchymal transition (EMT) signaling pathway and the mesenchymal to epithelial transition (MET) signaling pathway [82]. These transitions are important for metastasis as the EMT process allows cells to have increased migration and invasion, while the MET process helps with colony formation at new sites away from the primary tumor [82]. As seen in Table 1, in prostate cancer, cell lines PC-3M-1E8 and PC-3M-2B4 epithelial markers like E-cadherin were decreased, and mesenchymal markers like vimentin where increased when lamin A/C was knocked down [82]. Therefore, it is possible that the lowered expression in low and intermediate Gleason score tumors followed by the increase in lamin A/C in higher Gleason score tumors seen in other studies is associated with the MET process, which allows the cancer to form new colonies once it has metastasized [82]. 

Rather than expression levels, the positioning of both the *LMNA* gene and lamin A/C protein have been shown to be altered in prostate cancer [8,113]. Meaburn and Mistelli found that the *LMNA* gene frequently repositions more internally in the nucleus in non-metastatic cancer [8]. Therefore, they suggest that *LMNA* positioning could potentially be used to determine metastatic from non-metastatic cancers [8]. However, while the false positive rate for the assay they used to determine *LMNA* positioning was only 8.3%, the false negative rate was 42.9% [8]. 

As well as the gene’s position, the lamin A/C protein position is also altered in prostate cancer. As previously mentioned, in non-cancer cells, lamin B1 silencing causes nuclear blebs found to contain only lamin A/C and no B-type lamins [11,22,26]. Despite lamin B1 not being silenced in prostate cancer, prostate cancer cell lines frequently have similar blebs termed lamin B-deficient microdomains (LDMDs) [113]. LDMDs are enriched with RNA polymerase II and androgen receptor (AR) [113]. DHT (dihydrotestosterone) binds AR and plays a role in prostate cancer progression and LDMD frequency increases in the presence of DHT [113]. Frequency of LDMDs is correlated with Gleason score in tissue samples and increased motility in cell lines [113]. In addition, several chromosomal regions associated with prostate cancer are found to localize to LDMDs, and LDMDs contain mostly euchromatin [113]. However, despite this, genes localized to LDMDs show reduced expression, and it is possible that LDMDs actually serve a protective function against the progression of prostate cancer [113].

Progerin is expressed in PC3, DU145, and LNCaP cell lines [114]. As previously mentioned, while it causes senescence in normal cells, progerin does not cause senescence in tumor cells [74,114]. However, Tang et al. did find that overexpression of progerin in the PC3 cell line did result in an increased sensitivity to DNA damage [114]. Additionally, tumors from the transfected PC3 cells in nude mice grew faster than controls [114]. Therefore, it is possible that progerin, by increasing DNA damage without causing senescence, contributes to genomic instability of prostate cancer cells, therefore, resulting in more aggressive tumors [114]. 

## 18. Conclusions

With functions from maintaining the structural stability of the nucleus, cell motility, to DNA damage repair, lamin A/C is clearly an important protein in maintaining proper cell functioning. This is demonstrated by the fact that loss of lamin A/C results in lethality in mice and that mutation of the *LMNA* gene leads to a wide variety of laminopathies [13,15,67]. Lamin A/C also frequently plays a role in cancer, with lamin A/C being misregulated in a wide variety of cancers (Table 1, Table 2 and Table 3). As lamin A/C has so many functions and interacts with so many binding partners, as shown in Figure 1, determining what lamin A/C’s exact effect in cancer is, is difficult. As demonstrated by Table 1, Table 2 and Table 3, there are no clear patterns from lamin A/C expression in cancer, with some types overexpressing, while other types show under expression. Like other cancers, lamin A/C expression in prostate cancer is somewhat varied, and many studies have found contradictory results when it comes to how lamin A/C affects prostate cancer. Several studies have found that increased lamin A/C expression is associated with a higher Gleason score or a more aggressive phenotype in prostate cancer cell lines [81,111]. However, other studies have found what appears to be the opposite, with low lamin A/C levels leading to greater metastatic potential and poorer prognosis [7,78,112]. Additionally, rather than aberrant expression, aberrant lamin A/C organization in the nuclear lamina and progerin have also been shown to be involved in prostate cancer [113,114]. There may be many reasons for the lack of consensus on Lamin A/C effects on prostate cancer and other cancer’s, such as tumors showing heterogeneous lamin A/C expression, lamin A/C’s many roles in the cell and in cancer progression, and the fact that lamin A and lamin C expression levels can change independently of one another [70,71,72,81,82,111]. While lamin A/C could potentially be a useful biomarker and allow for more accurate diagnosis and treatment of prostate cancer and many other cancers, more research is still needed to fully elucidate lamin A/C’s role in cancer.

## Figures and Tables

**Table 1:** Lamin A/C expression effect on different cancer cell lines.

Cell and Cancer Type	Lamin A/C Expression	Effect on Cancer	Reference
Lung cancer cell lines A549, and H1299	Decrease in lamin A/C	Increased tumor senescence	[73]
Human non-small cell lung carcinoma H 1299, human osteosarcoma U-2 OS, human cervix carcinoma HeLa	Lamin A/C mutated to S22A-progerin	Increased cellular senescence	[74]
Breast adenocarcinoma cell lines MDA-MB-231 and MDA-MB-468	Lamin A/C knockout	Decreased cell’s resistance to fluid shear stress	[75]
Human pre-metastatic colon adenocarcinoma cell line SW480	Transfected with GFP-lamin A	Downregulated E-cadherin and increased cell motility	[76]
Human pre-metastatic colon adenocarcinoma cell line SW480	Lamin A/C upregulated	Increased cell motility	[65]
Human ovarian cancer cell line H08910	Lamin A/C overexpressed, Lamin A/C 30–40% inhibited, lamin A/C 70–80% inhibited	Lamin A/C overexpressed: Decreased migration through 3 μm pore. 30–40% inhibited: Increased migration through 3 μm pore. 70–80% inhibited: Decreased migration through 3 μm pore	[77]
Prostate cancer cell line DU145 and breast cancer cell line BT-549	Lamin A/C depleted	Emerin mislocalized and nuclear membrane had blebbing	[78]
Neuroblastoma cell line SH-SY5Y	Lamin A/C downregulated	Tumor initiating cells developed	[79]
Breast cancer cell line MDA-MB-231 in suspension culture	Lamin A/C downregulated	Decreased adhesion and reattachment of cells	[80]
Prostate cancer cell line PC3, DU145, and LNCaP	Lamin A/C upregulated	Increased PI3k subunit, p110, and p85 expression and increased growth and invasive capabilities	[81]
Prostate cancer cell line PC-3M-1E8 and PC-3M-2B4	Lamin A/C knockdown	Cell growth was inhibited and colony formation was decreased. E-cadherin downregulated. Vimentin, snail, and slug upregulated	[82]

**Table 2:** Lamin A/C expression found in patient samples.

**Cancer Type**	**Lamin A/C Expression**	**Reference**
56 invasive ductal carcinoma samples	Majority of samples heterogeneous for lamin A/C expression, 38% had no lamin A/C expressed	[83]
656 colorectal adenocarcinoma tumor samples	70% positive for lamin A/C, 30% negative	[76]
73 breast cancer tumor samples	84.9% had low lamin A/C expression (low defined as less than 50% of cancer cells stained positive for lamin A/C)	[84]
33 small cell lung carcinomas (SCLC), 72 non-small cell lung carcinomas (34 adenocarcinoma, 30 squamous cell carcinoma, 8 large cell carcinoma)	91% of SCLC had negative or low lamin A/C expression, 3% of non-SCLC had negative or low lamin A/C expression	[85]
115 breast cancer tissue samples	Lower lamin A/C than found in non-cancerous breast tissue.	[86]
126 gastric carcinoma samples	70 positive for lamin A/C 56 negative for lamin A/C (lamin A/C associated with poorer prognosis and lower differentiation)	[87]
87 endometrial cancer samples	Lamin A was reduced in all high-grade endometrial cancer samples	[88]
61 epithelial ovarian cancer samples	Lower lamin A expression than normal and benign controls	[70]
17 primary colorectal carcinomas, 18 adenomatous polyps	Lamin A/C reduced or absent in all samples	[66]
128 breast adenocarcinomas	Lamin C expression increased, Lamin A expression decreased	[71]
76 cervical uterine smears (CUS)	39% normal expression, 28% weak, 33% none. Oncogenic HPV infection rate highest in group with no lamin A/C staining.	[89]
219 stage II and 151 stage III colon cancer samples	17.8% low lamin A/C expression, Reoccurrence of cancer 45.5% in low lamin A/C expression group compared to 29.6% in high lamin A/C expression group	[90]
94 prostate tumor tissue microarrays	Lamin A/C had low expression in tumor regions with a Gleason pattern (GP) less than 3 and higher expression in regions with a GP of 4 or 5	[81]
4 prostate adenocarcinoma cohorts	Lamin A/C mRNA reduced when the Gleason score is 8, but the level increases above Gleason score 8 and in metastatic regions	[82]
Tissue microarray from 501 prostate cancer patients	Low lamin A/C associated with increased lymph node metastasis and disease-specific death	[7]
Tissue cores from 94 prostate tumor samples	Lamin A expression higher in higher Gleason score tumors	[91]
Biopsies from 9 patients with Hodgkin’s disease	Most Reed-Sternberg and Hodgkin cells expressed lamin A/C while surrounding B and T lymphocytes did not.	[92]

**Table 3:** Lamin A/C expression effect on different cancers in patient samples.

Cancer Type	Lamin A/C Expression	Effect on Cancer	Reference
Invasive breast carcinoma	Reduced lamin A/C expression	More aggressive phenotype than tumors with high lamin A/C expression	[93]
Stage II and Stage III colon cancer tumors	Low lamin A/C expression	Increased disease recurrence	[90]
Colorectal adenocarcinoma tumors	Sample either did or did not express lamin A/C	Mortality risk twice as high as tumors not expressing lamin A/C	[76]
